# Early Detection of Bronchial Lesions Using Lung Imaging Fluorescence Endoscope

**DOI:** 10.1155/DTE.5.85

**Published:** 1999

**Authors:** N. Ikeda, H. Honda, T. Katsumi, T. Okunaka, K. Furukawa, T. Tsuchida, K. Tanaka, T. Onoda, T. Hirano, M. Saito, N. Kawate, C. Konaka, H. Kato, Y. Ebihara

**Affiliations:** 1 Department of Surgery Tokyo Medical University 6-7-1, Nishishinjuku Shinjuku-ku Tokyo 160-0023 Japan; 2 Department of Pathology II Tokyo Medical University 6-7-1, Nishishinjuku Shinjuku-ku Tokyo 160-0023 Japan

## Abstract

The performance of the Lung Imaging Fluorescence Endoscope (LIFE) system was compared with conventional bronchoscopy in 158 patients: 68 patients with invasive cancer, 42 patients with abnormal sputum cytology findings (12 early cancer and 26 dysplasia), 17 cases with resected lung cancer and 31 smokers with symptoms. The respective results of conventional bronchoscopy and LIFE for detection of dysplasia were; sensitivity 52% and 90% (biopsy basis), 62% and 92% (patient basis). Fluorescence bronchoscopy may be an important adjunct to conventional bronchoscopy to improve the localization of subtle lesions of bronchus.


*Diagnostic and Therapeutic Endoscopy*, Vol. 5, pp. 85–90
Reprints available directly from the publisher
Photocopying permitted by license only

© 1999 OPA (Overseas Publishers Association) N.V.
Published by license under
the Harwood Academic Publishers imprint,
part of The Gordon and Breach Publishing Group.
Printed in Singapore.

### Early Detection of Bronchial Lesions Using Lung Imaging Fluorescence Endoscope

N. IKEDA[a,*], H. HONDA[a], T. KATSUMI[a], T. OKUNAKA[a], K. FURUKAWA[a], T. TSUCHIDA[a], K. TANAKA[a], T. ONODA[a], T. HIRANO[a], M. SAITO[a], N. KAWATE[a], C. KONAKA[a], H. KATO[a] and Y. EBIHARA[b]

[a]*Department of Surgery,* [b]*Department of Pathology II, Tokyo Medical University, 6-7-1, Nishishinjuku, Shinjuku-ku, Tokyo 160-0023, Japan*

**The performance of the Lung Imaging Fluorescence Endoscope (LIFE) system was compared with conventional bronchoscopy in 158 patients: 68 patients with invasive cancer, 42 patients with abnormal sputum cytology findings (12 early cancer and 26 dysplasia), 17 cases with resected lung cancer and 31 smokers with symptoms. The respective results of conventional bronchoscopy and LIFE for detection of dysplasia were; sensitivity 52% and 90% (biopsy basis), 62% and 92% (patient basis). Fluorescence bronchoscopy may be an important adjunct to conventional bronchoscopy to improve the localization of subtle lesions of bronchus.**

*Keywords:* Fluorescence bronchoscopy, Early detection, Early lung cancer, Dysplasia

### INTRODUCTION

The increasing numbers of lung cancer deaths are mainly due to the detection of the disease at a late stage. Prevention and early detection are essential to decrease the death rate. When roentgenologically occult lung cancer is detected by sputum cytology, the only tool to localize the lesion is endoscopy. However carcinoma *in situ* can be difficult to identify by conventional white light bronchoscopy alone [1]. Also, the endoscopic apperance of dysplasia is so subtle that even experienced bronchoscopists sometimes fail to localize the lesions. To solve this problem, fluorescence diagnosis was applied to endoscopic imaging by Profio [2]. Kato *et al.* began a clinical trial using fluorescence bronchoscopy, employing a tumor-specific photosensitizer as a sensitive and accurate method to detect central type early lung cancer in 1980 [3–5]. Another approach has been made by Palcic and Lam who noticed the difference of autofluorescence between normal and tumor tissue. Using a high quality CCD and special algorithm, they developed the LIFE system [6,7]. As previously reported [8,9],

* Corresponding author. Tel.: +81 3 3342 6111. Ext. 5071. Fax: +81 3 3349 0326.

this diagnostic procedure is helpful to detect subtle lesions in the bronchus which can hardly be detected by conventional bronchoscopy.

In this study, the authors will determine if fluorescence bronchoscopy using the LIFE system could improve the diagnostic rate of bronchial lesions, especially early cancer and dysplastic lesions, compared with conventional bronchoscopy.

### MATERIALS AND METHODS

A total of 158 subjects were studied from June 1997 to May 1998 in Tokyo Medical University Hospital and 262 sites were biopsied. The criteria for enrollment in this study were as follows:

(1) Patients with cancer who were scheduled for endoscopic examination before treatment (68 cases).
(2) Patients with abnormal sputum findings with normal chest X-ray (42 cases).
(3) Patients who had undergone curative operation of stage I lung cancer scheduled for endoscopy for periodical follow-up (17 cases).
(4) Smokers with respiratory symptoms (31 cases).

The detailed characteristics of cases are shown in Table I.

Conventional fiberoptic bronchoscopy (BF-20, Olympus Optical Co., Tokyo, Japan) was first performed and areas with abnormal findings (suspicious for cancer or dysplasia) were noted for subsequent biopsy. The fluorescence examination using LIFE (Xillix Technologies Corp, Richmond, B.C., Canada) was then performed by the same endoscopy team. The details of the LIFE system have been reported previously [6,8]. Normal areas show green autofluorescence when excited by blue light but abnormal areas showed decreased autofluorescence (Fig. 1), therefore, abnormal areas were indicated by cold spots under fluorescence imaging [7,10]. Any abnormal areas detected by conventional bronchoscopy or by the LIFE device were biopsied. Biopsy procedure was performed under fluorescence imaging if the suspicious areas could not be identified by conventional bronchoscopy. Procedures were carried out under local anesthesia and no additional sedation was necessary. All biopsy specimens were interpreted in our Department of Pathology and pathological diagnosis was the gold standard for the diagnosis of the subjects as well as for the comparison of the diagnostic accuracy of conventional bronchoscopy and fluorescence diagnosis.

TABLE I Characteristics of enrolled patients (158 cases)

| | |
|---|---|
| Lung cancer | 68 cases |
| endoscopically recognized/unrecognized | 43/25 |
| squamous cell carcinoma | 30 |
| adenocarcinoma | 27 |
| small cell carcinoma | 4 |
| large cell carcinoma | 3 |
| others | 4 |
| Abnormal sputum cytology findings | 42 cases |
| early cancer | 12 |
| dysplasia | 22 |
| origin unknown | 10 |
| Follow up after operation | 17 cases |
| Smokers with symptoms | 31 cases |

### RESULTS

There were 112 men and 46 women with a mean age of 64 (range 33–78). Of those, 132 (84%) were current or former smokers with a mean smoking index of 840. The final pathological diagnosis of 262 biopsy specimens were as follows: normal, inflammation, metaplasia with no atypia in 135; dysplasia in 72; early cancer in 12; and invasive cancer in 43.

### Biopsy-based Analysis

Of the 262 biopsies, cancer was detected at 55 sites (invasive cancer 43, early cancer 12) (Table II). Both conventional bronchoscopy and the LIFE system correctly diagnosed these lesions, but the extent and margin of the lesions were more objectively observed under fluorescence examination. Dysplasia was detected in 72 sites, 19 sites were detected in invasive cancer patients, 10 from early cancer, 36 sites from subjects with abnormal sputum findings,

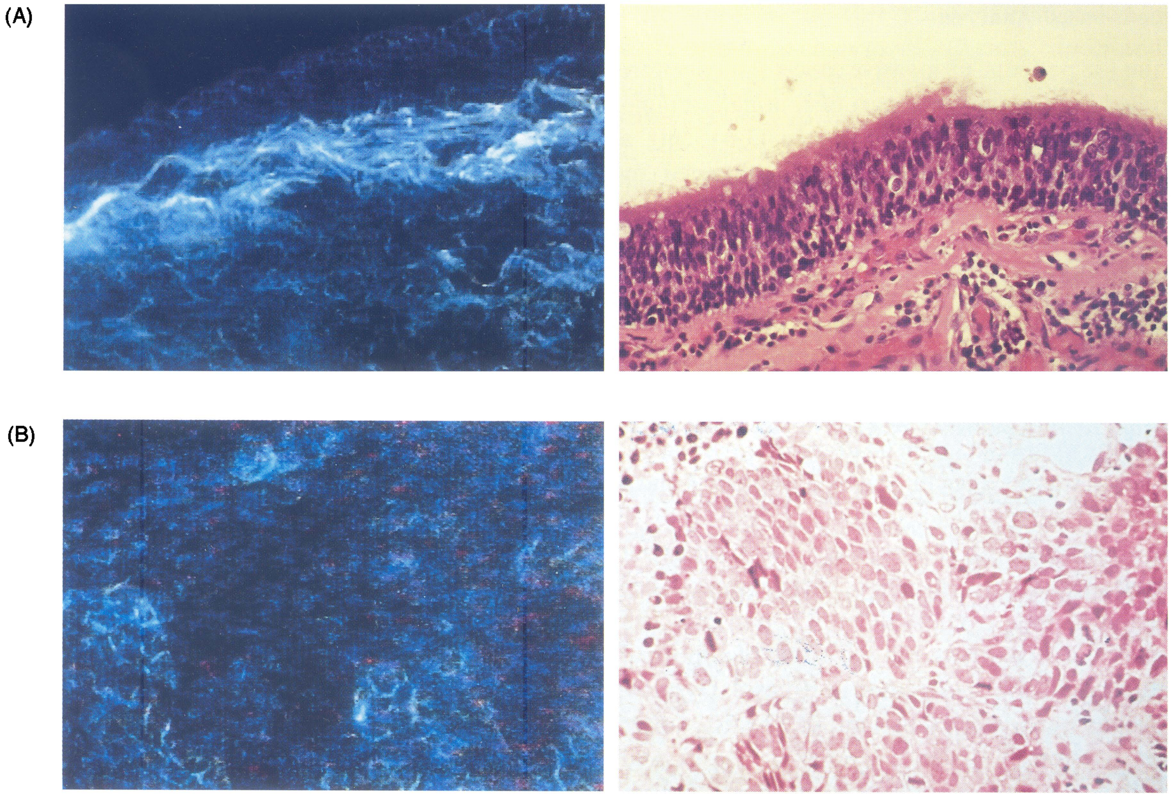

FIGURE 1 Finding of normal bronchus (A) and the tumor (B) by fluorescence microscopy. Submucosal layer of normal bronchus has strong autofluorescence excited at 420 nm, while tumor tissue has lost autofluorescence.

TABLE II Results of conventional bronchoscopy and the LIFE system (Biopsy-based analysis, $n = 262$)

| | LIFE negative | LIFE positive |
|---|---|---|
| Invasive cancer ($n = 43$)* | | |
| BF positive | | 43 (100%) |
| Early cancer ($n = 12$) | | |
| BF positive | | 12 (100%) |
| Dysplasia ($n = 72$) | | |
| BF negative | 0 (0%) | 35 (49%) |
| BF positive | 7 (10%) | 30 (42%) |
| Normal/Inflammation ($n = 135$) | | |
| BF negative | 60 (44%) | 24 (18%) |
| BF positive | 30 (22%) | 21 (16%) |

* Endoscopically unrecognized cases (25 cases) were excluded from analysis.

TABLE III The prevalence of dysplasia

| | No. of cases | No. of sites | No. of cases with multiple sites |
|---|---|---|---|
| Group I ($n = 68$) | 12 (18%) | 19 | 5 (7%) |
| Group II ($n = 42$) | 24 (57%)* | 36 | 12 (29%) |
| Group III ($n = 17$) | 4 (24%) | 5 | 1 (6%) |
| Group IV ($n = 31$) | 2 (7%) | 2 | 0 (0%) |

I, lung cancer; II, abnormal sputum cytology findings; III, follow up after operation; IV, smokers with symptoms.
* 4 cases (10 sites) with early cancer.

5 from post-operative follow up, and 2 from smokers with recurrent symptoms (Table III). The sensitivity for dysplasia detection were 51% by conventional bronchoscopy and 90% by the LIFE system. The positive predictive value for dysplasia and cancer was 64% by conventional bronchoscopy and 73% by the LIFE system. In relation to specificity, conventional bronchoscopy showed 62% and LIFE 66% (Table V).

### Patient-based Analysis

Of the 158 patients, 68 invasive cancer patients were enrolled in this study as a part of routine examination before treatment (Table IV). In 43 patients, a definitive diagnosis of cancer was obtained by bronchial biopsy and these lesions were observed both by conventional and fluorescence bronchoscopy. In the rest of 25 patients, the primary lesions were located in the periphery of the lung, and could not be observed endoscopically. These lesions were diagnosed by transbronchial lung biopsy (TBLB) or percutaneous needle cytology. A total of 19 dysplastic lesions were detected in 68 invasive cancer cases, one lesion was detected in 7 cases, 2 lesions in 4 and 4 lesions in 1 case.

In 42 patients with abnormal sputum findings but normal chest X-ray, central type early squamous cell carcinoma was diagnosed in 12 patients and dysplasia in 20 patients, but the origin of abnormal sputum could not be identified in 10 cases. Dysplastic lesions were detected in 4 out of 12 early cancer cases; 1 lesion in 1 case, 2 lesions in 2 cases, and 5 lesions in 1 case. A total of 36 dysplastic lesions were detected in 20 dysplasia cases; 1 lesion in 11 cases, 2 lesions in 6 cases, 3 lesions in 1 case, 4 lesions in 1 case, and 6 lesions in 1 case. Endoscopic localization of the foci of 42 cases of abnormal sputum cytology findings was successful in 24 cases (57%, 12 early cancer and 12 dysplasia) by conventional bronchoscopy and in 32 cases (76%, 12 early cancer and 20 dysplasia) by the LIFE system.

TABLE IV Results of conventional bronchoscopy and the LIFE system (patient basis analysis, $n = 158$)

| | LIFE negative | LIFE positive |
|---|---|---|
| Invasive cancer ($n = 68$)* | | |
| BF positive | | 43 (100%) |
| (TBLB, Needle biopsy 25) | | |
| Early cancer ($n = 12$) | | |
| BF positive | | 12 (100%) |
| Dysplasia ($n = 26$) | | |
| BF negative | 0 (0%) | 10 (38%) |
| BF positive | 2 (8%) | 14 (54%) |
| Normal/Inflammation ($n = 52$) | | |
| BF negative | 15 (29%) | 8 (15%) |
| BF positive | 12 (23%) | 17 (33%) |

* Endoscopically unrecognized cases (25 cases) were excluded from analysis.

Among post-operative 17 cases, 4 cases (24%) were revealed to have dysplastic lesions (1 lesion in 3 cases and 2 lesions in 1 case), of which 2 cases were diagnosed only by fluorescence examination. No recurrent case was found in those patients. In the group of smokers with continuous or recurrent symptoms (31 cases), 2 cases (6%) were diagnosed to have dysplasia. The prevalence of dysplasia is shown in Table III. The results of patient basis analysis are shown in Table IV.

Sensitivity of dysplasia detection by patient based analysis was 62% by conventional bronchoscopy and 92% by the LIFE system. There were 52 patients who were pathologically diagnosed as having chronic inflammation or found to be normal. Chronic inflammation was proved by findings of biopsy specimens such as reserve cell hyperplasia, thickened basement membrane, or lymphoid infiltration, etc. Specificity by patient based analysis was 44% for conventional bronchoscopy and 52% for the LIFE system (Table V).

### DISCUSSION

Sputum cytology examination is often the first method to detect central type early cancer or dysplasia, but even experienced bronchoscopists sometimes fail to localize lesions. Such lesions are too thin to show endoscopically visible mucosal changes [1]. Dysplasia has been regarded as a

TABLE V Summary of clinical data

| | Biopsy basis | | Patient basis | |
|---|---|---|---|---|
| | BF (%) | LIFE (%) | BF (%) | LIFE (%) |
| Sensitivity | | | | |
| cancer and dysplasia | 72 | 95 | 88 | 98 |
| dysplasia | 51 | 90 | 62 | 92 |
| Positive predictive value | 64 | 73 | 71 | 76 |
| Specificity | 62 | 66 | 44 | 52 |

*Note*: Cancer cases with invisible primary lesions were excluded from analysis.

precancerous state of central type early cancer according to the concept of carcinogenesis as a multistep process [11,12]. Dysplasia can be found in patients with non-cancerous disease, therefore the relationship between cancer and dysplasia is not well confirmed. However, if dysplasia is a precancerous stage, diagnosis of dysplasia would be helpful for the prevention or early detection of lung cancer. The bronchoscopic findings of dysplasia have not been carefully studied. Thickening of the mucosa is a common finding of dysplasia but such a finding is also observed in inflamed airways. Lam and Palcic noticed the lack of autofluorescence in the tumor lesion and they amplified the difference of autofluorescence between normal and tumor tissue for clinical use. Using a high quality CCD, image intensifier and special algorithm, the LIFE system was developed [6–8]. As previously reported, the diagnostic procedure is helpful to detect subtle lesions in the bronchus which can hardly be recognized by conventional bronchoscopy [8,9]. Lam and associates reported the result of a multicenter clinical trial in which 173 patients were enrolled. The relative sensitivity of white light with LIFE vs white light alone was 6.3 for intraepithelial lesions. The positive predictive value was 0.33 and 0.39 and the negative predictive value was 0.89 and 0.83 [13]. In the present study, the sensitivity of conventional bronchoscopy for dysplasia was 51% and that of the LIFE system was 90%. The positive predictive value for dysplasia or cancer by conventional bronchoscopy was 64% and that by the LIFE system was 73%.

Since invasive cancer is usually visible, the aim of fluorescence diagnosis is not to localize invasive cancer but to detect early lesions which could not be observed by conventional bronchoscopy. We detected 12 early cancer in this study, all of which could be observed under conventional bronchoscopy as well. At this stage, to observe the extent of tumor is one goal of the LIFE system in cancer management. The LIFE system, when used as an adjunct to standard bronchoscopy, is a powerful device to detect dysplastic lesions. A total of 42 subjects with abnormal sputum cytology findings were enrolled in this study. Localization was successful in 24 cases (57%, 12 early cancer and 12 dysplasia) by conventional bronchoscopy and in 30 cases (71%, 12 early cancer and 18 dysplasia) by the LIFE system. Dysplastic lesions were detected in 18% of invasive cancer cases, 33% in CIS cases, 24% in post-operative cases and 7% in smokers with symptoms.

We postulated that the phenomenon of a high frequency of dysplasia in cancer cases is related to field cancerization, therefore cases with dysplasia should be periodically followed up as high risk cases. Auerbach and co-workers reported in their autopsy study that CIS was found in 15% of the subjects. Also they reported CIS was found in 4.4–11.4% in smokers [14]. As lung cancer patients tend to have synchronous or metachronous double cancer, endoscopic examination may be necessary even for cases which received operation for peripheral type adenocarcinoma.

The specificity of conventional bronchoscopy was 62% and that of the LIFE system was 66%. False positive cases in conventional bronchoscopy were mainly due to thickened bifurcations with normal mucosa and the reasons for false positive results with the LIFE system was mainly due to chronic inflammatory findings (reserve cell hyperplasia, thickened basement membrane) which showed cold spots under fluorescence imaging. The reason for suspicious LIFE findings in areas with normal histology is unknown. Molecular genetic changes such as cumulative gene losses during the progression of precancerous lesions have been reported [15]. The fluorescence image may reflect some molecular events which are not observed microscopically.

Fluorescence studies are spreading throughout the world, but some problems remain. It is known that there will be some disagreement concerning the diagnosis among pathologists particularly when the specimen is very small. Since it is impossible to distinguish carcinoma *in situ* from dysplasia using the LIFE system, the histopathology of biopsies is the gold standard. To evaluate the effectiveness of fluorescence studies, more detailed and objective criteria of histopathology of different degrees of dysplasia and carcinoma *in situ* should be established.

Due to increasing numbers in early cancers as well as more elderly patients, minimally invasive treatment is required to maintain quality of life. As previously reported, surgery can be avoided in some of the *in situ* and early cancer when treated by photodynamic therapy [16,17] while chemoprevention might reverse dysplasia to normal status [18].

At present it has been confirmed that fluorescence examination can be effective to increase the rate of detection of early lesions which can be treated by endoscopic treatment.

### Acknowledgement

The authors thank Professor J.P. Barron, International Medical Communications Center, Tokyo Medical University, for his excellent support in reviewing this manuscript.

### References

[1] Woolner, L.B., Fontana, R.S., Cortese, D.A. *et al.* Roentogenographically occult lung cancer: Pathologic findings and frequency of multicentricity during a 10-year period. *Mayo. Clin. Proc.* 1984; **59**: 453–466.

[2] Balchum, O.J., Doiron, D.R. and Profio, A.E. Fluorescence bronchoscopy for localizing bronchial cancer and carcinoma *in situ*. *Recent Res. Cancer Res.* 1982; **82**: 97–120.

[3] Kato, H. and Cortese, D.A. Early detection of lung cancer by means of hematoporphyrin derivative fluorescence and laser photoradiation. *Clin. Chest Med.* 1985; **6**: 237–253.

[4] Kato, H., Imaizumi, T., Aizawa, K. *et al.* Photodynamic diagnosis in respiratory tract malignancy using an excimer dye laser system. *J. Photochem. Photobiol.* 1990; **6**: 189–196.

[5] Okunaka, T., Furukawa, K., Tanaka, K. *et al.* Fluorescence photodiagnosis for malignant tumor. *SPIE* 1996; **2675**: 99–104.

[6] Palcic, B., Lam, S., Hung, J. *et al.* Detection and localization of early lung cancer by imaging techniques. *Chest* 1991; **99**: 742–743.

[7] Hung, J., Lam, S., LeRiche, J.C. *et al.* Autofluorescence of normal and malignant bronchial tissue. *Laser Surg. Med.* 1991; **11**: 99–105.

[8] Lam, S., MacAulay, C., Hung, J. *et al.* Detection of dysplasia and carcinoma *in situ* using a lung imaging fluorescence endoscope (LIFE) device. *J. Thorac. Cardiovasc. Surg.* 1993; **105**: 1035–1040.

[9] Ikeda, N., Kim, K., Okunaka, T. *et al.* Early localization of bronchogenic cancerous/precancerous lesions with lung imaging fluorescence endoscope. *Diagnostic and Therapeutic Endoscopy* 1997; **3**: 197–201.

[10] Furuya, T., Ikeda, N., Okada, S. *et al.* Autofluorescence of bronchial tissue. *J. Japan Society for Laser Medicine* 1996; **17**: 75–80 (in Japanese).

[11] Hirano, T., Franzen, B., Kato, H. *et al.* Genesis of squamous cell lung carcinoma sequential changes of proliferation, DNA ploidy, and p53 expression. *Am. J. Pathol.* 1994; **144**: 296–302.

[12] Konaka, C., Auer, G., Nasiell, M. *et al.* Sequential cytomorphological and cytochemical changes during development of bronchial carcinoma in beagle dogs exposed to 20-nethylcholanthrene. *Acta Histochem. Cytochem.* 1982; **15**: 779–797.

[13] Lam, S., Kennedy, T., Unger, M. *et al.* Localization of bronchial intraepithelial neoplastic lesions by fluorescence bronchoscopy. *Chest* 1998; **113**: 696–702.

[14] Auerbach, O., Stout, A.P., Hammod, E.C. *et al.* Changes in bronchial epithelium in relation to cigarette smoking and in relation to lung cancer. *N. Engl. J. Med.* 1961; **265**: 253–267.

[15] Thiberville, L., Payne, P., Vielkinds, J. *et al.* Evidence of cummulative gene losses with progression of premalignant epithelial lesions to carcinoma of bronchus. *Cancer Res.* 1995; **55**: 133–139.

[16] Kato, H., Okunaka, T. and Shimatani, H. Photodynamic therapy for early stage bronchogenic carcinoma. *J. Clin. Laser Med. Surg.* 1996; **14**: 235–238.

[17] Hayata, Y., Kato, H., Konaka, C. *et al.* Photodynamic therapy in early stage lung cancer. *Lung Cancer* 1993; **9**: 287–294.

[18] Saito, M., Kato, R, Tsuchida, T. *et al.* Chemoprevention effects on bronchial squamous metaplasia by folate and vitamin $B_{12}$ in heavy smokers. *Chest* 1994; **2**: 496–499.

